# The Application of Some Principles of Law to the Practice of Dentistry

**Published:** 1895-07

**Authors:** W. A. Purrington

**Affiliations:** New York


### THE APPLICATION OF SOME PRINCIPLES OF LAW TO THE PRACTICE OF DENTISTRY.[1]

BY W. A. PURRINGTON, ESQ., NEW YORK.[2]

MR. PRESIDENT AND MEMBERS OF THE ODONTOLOGICAL SOCIETY,—It would be impossible for me to treat at length the topics enumerated in the syllabus of this paper unless I should read to you a volume. The most that I can hope to do is to present with a little detail what is most interesting to you, the legal status and regulation of your profession, and to treat other topics with a very broad brush. It is a fundamental principle that every man should be allowed to earn his livelihood by following any calling for which he is fitted and in which he gives satisfaction to those employing him; the individual, as a rule, knowing much better than the State what is good for him and the value of services rendered by and to him. It is also an accepted principle that, under the police powers, the State has a right to make general laws for the punishment of fraud and imposture, and to protect the general health and welfare. But it is no business of the State to interfere with the private concerns of the citizen in order to secure for him a monopoly in business, to protect him from competition, to increase his dignities, or to do for his individual advantage anything that is not necessary, or, at least, conducive to the common welfare. These are trite sayings, yet they cannot be too often emphasized; for, like many other formulæ, they may be learned by rote with little appreciation of their full significance.

*The question of limiting by license laws the right to practise medicine* has been discussed hotly for very many years. When the people are bent on following certain courses, or employing certain persons, or entertaining certain opinions, or reading certain books, legislation cannot stop them. The laws of England, the power of the College of Physicians, the decisions of the Court of King's Bench, all failed to suppress the recalcitrant apothecary Rose, who dared to prescribe a bolus to Searle, the butcher, in defiance of the physicians and the law. Beaten in the law courts, Rose took his case into the House of Lords, and there obtained a decision in his favor, which, however illogical in law, was entirely comprehensible from the stand-point of those who saw only an intolerable burden in a system

---

[1] Read before the New York Odontological Society, February 19, 1895.

[2] Counsel of the Dental Society of the State of New York.

that forced a man to pay physician, surgeon, and apothecary in the same case for services that any one of them could render except for legal prohibition.

In this State of New York the physicians early organized societies and easily controlled the practice of medicine until they undertook forcibly to crush out homœopathy by social and professional ostracism, objurgation, and such implements of persecution as the civilization of our time permits. Instead of laughing at the bizarre, metaphysical ideas of Hahnemann, the doctrines of high potencies and itch, which few of his avowed disciples now pretend to believe; instead of leaving every intelligent physician free not only to investigate the doctrine but to accept what good, if any, might be in it while throwing aside its chaff, its opponents treated the new teaching as medical heresy; and heresy is a plant that flourishes best when watered with the rain of persecution. A silly doctrine that would wither in the brightness of a laugh and die in the clear light of demonstration can sprout and bear fruit under the shadow of oppression. And the net result of the crusade against homœopathy in this State has been that, instead of recognizing as physicians only those with sufficient education to fit them for proving all medical theories and holding fast the truth as they see it, without blind subservience to what has been taught in the past, the statute law of the State of New York has recognized for years an absurd division of medical men into physicians, homœopathic physicians, and eclectic physicians; and we have the stars to thank that Thompsonians, who did once get a slight footing in the statute book, vitopaths, hydropaths, and all sorts of other paths, voudooists, faith healers, Christian scientists, and the whole quacking flock, have not been assigned separate coops in the barn-yard of our statute book.

In 1844 sympathy for the homœopaths effected such a modification of the medical laws that for years thereafter the unlicensed practitioner of medicine was guilty of misdemeanor only in case of gross malpractice. In other words, just as it is said in law that every dog is entitled to one bite before he can be adjudged dangerous, so, under this statute, every unlicensed practitioner was entitled to kill or maim somebody before he could be considered a misdemeanant.

Not only did physicians attempt to control theories of medicine by treating heretics as quacks amenable to legal punishment, but their societies also undertook to establish fee bills, in trades-union fashion, and to treat the acceptance of less than the tariff fee as unprofessional conduct. When, however, the Medical Society of Erie tried to expel a certain Dr. Gray for treating the county poor at less than prescribed rates, the Supreme Court decided that the corporation had transcended its powers by enacting regulations contrary to public policy.

*The constitutionality of laws* prohibiting the practice of medicine to persons not qualified under the statute has been affirmed by the courts of almost every State of our Union, and it has been held that the right to practise medicine is not property in such a sense that it is taken away without due process of law by regulative statutes, providing that they are general in their application and do not favor one individual more than another. The controlling decision on this point is that of the Supreme Court of the United States in the case of Dent *vs.* State of West Virginia (129 U.S., 114). And it is now established that a legislature has power to make at any time a uniform law with which every practitioner of medicine, no matter what his standing or how long he may have been in practice, must comply before he can continue to follow his profession. Such a statute was our own Medical Registration Law of 1880; and although its requirement that all practitioners should register was no hardship, nevertheless there were physicians of good standing who, feeling aggrieved that after years of practice in the community they should be compelled to register with the county clerk as a condition of continuing their business, flatly refused to obey the law until persuaded to do so by the issuance from the police courts of warrants for their arrest.

Up to this point I have spoken of medical laws, partly because they have set the pace, partly because I treat dentistry as a specialty of medicine; and the same reasoning upon which medical laws are held to be constitutional applies equally to those regulating dental practice. The desirability and efficacy of a law are, however, quite apart from its constitutionality. Many laws are constitutional that are not desirable; some, which are constitutional and would be desirable if efficacious, are so repugnant to common habits and feelings as to be almost entirely unenforcible. Whether legislation such as we are discussing is desirable depends upon the point of view. It is certainly desired by some as a means of limiting competition and enhancing the fees of individuals. *If that were its sole purpose I should be the first to urge that the statute book be purged of it.* If the sole fruit of such laws were the conviction and punishment of a few score of miserable offenders, I should say that money could not be worse spent than in enforcing them. The only justification of legislation that restricts man's liberty to earn his bread is that it is needed to protect the public. How do medical and dental laws operate to achieve this end? I think in a twofold fashion. First, by requiring that licentiates shall have pursued an adequate course of study, they, in a large measure, protect the citizen against the ignorant quack, although powerless, more is the pity, to protect him against a quack of education. Second, such laws are schoolmasters with three classes of pupils. They teach the public that the care of the body should not be, certainly need not be, intrusted to persons with no knowledge of its functions or the means of relieving its disorders, and by the registration system they afford a simple way of ascertaining whether a practitioner has conformed to the requirements of the law. Again, they teach the unlicensed practitioner that he must walk warily. Lastly, they teach the licentiate himself that knowledge is the foundation of his career; and rightly understood and applied, they cultivate in him the professional motive which, even if self-seeking, as all human motives are in some degree, is, nevertheless, higher than that embodied in the trade maxim, *caveat emptor.* In theory, then, the law is justified to the public at large because it protects the citizen, while to the professional man it has the added but incidental merit of being an aid in maintaining the honorable standard of his calling.

*As to the efficacy of such legislation* for these purposes, you are each and all qualified as well as, or better than, I to speak. I can but tell of the record of convictions: you from your daily contact with your colleagues, your students, and your patients, know what effect the law has in preventing the ignorant from entering your ranks and the public from suffering at their hands. The mere existence of a law on the statute book is of little avail to effect its purpose: "'tis the eye of childhood that fears a painted devil." In order that it may be feared a law must be enforced, firmly and surely, but also equably and in a broad spirit, lest injustice masquerade in the ermine of the law. And here is the rub. How is this to be done? If the enforcement of these laws were left to the ordinary machinery of justice,—the police magistrates, grand juries, and district attorneys,—a conviction of an illegal practitioner of medicine or dentistry would be as "rare as a day in June." This is no reflection on the officers of the law. On the contrary,—and it gives me very great pleasure to say this at this moment,—all law officers in this county with whom I have had to deal have done their full duty under these statutes. But they have enough to do in detecting and punishing the offences that are criminal *per se*, the great crimes against God, nature, and civilization, murder, robbery, arson, swindling, and assault, crimes which the injured hasten to report and avenge. And it is not possible under existing conditions for these officers, at least not in great cities, to detect the numberless mere statutory offences, so hidden away in dark corners of the law that none of us know all of them and all of us may be guilty of some of them. Probably there is not a father here to-night who knows that if his tender-hearted boy or girl should find an English sparrow at the window on these frosty mornings and give the bird a crumb, the child, if of age of discretion, would be guilty of a misdemeanor under Chapter 641 of the laws of 1887. Fortunately, the enthusiasts who put that law on the statute book did not organize a "Society for the Prevention of Kindness to Sparrows," employing zealous agents and trained sparrows to secure evidence; and our children may, therefore, notwithstanding this law, follow, in comparative safety, their humane instincts and commit the misdemeanor of giving food and shelter to the *passer domesticus* without much likelihood of interference from the police. All our sanitary laws, laws for the protection of children and animals, and generally all laws prohibiting acts to the punishment of which their victims are not spurred on by revengeful motives, must either be enforced by private organizations or else, falling into common oblivion, become hidden traps for the unwary.

The road of a prosecuting witness is thorny; he must go before police magistrates and grand and petit juries until, except in the graver crimes, he is fain to let the offender go unwhipped rather than sacrifice more of his time and comfort. When it comes to enforcing medical and dental laws there are other difficulties than the ordinary reluctance of a witness to appear in court against the offender. Often the patient is friendly or sympathetic and therefore loath to testify, or he is chiefly bent on getting back money paid for possibly adequate services, and the chances are that if not actually bought off, his testimony will be unsatisfactory. It becomes necessary, therefore, to employ agents. And here we are confronted with two dangers: First, the danger of blackmailing; for the agent has it in his power to report an offender or not to do so; and this opportunity for oppression is, to my mind, the greatest danger and evil of all statutes enforced in this fashion. Secondly, the danger that, with an honest agent, the zeal of his work will so consume him that, in order to make a record, he will entice men into committing the very crime for which he will demand their punishment. To prevent such evil doings is the best service that counsel to such societies can render. Nothing could be more foolish than to suppose that a long list of convictions is the best proof of a wise enforcement of the statute. It would be easy for reckless or unscrupulous men to fill the morrow's police court with technical offenders against medical and dental laws. But a few such performances would work the repeal of the statute. And so in enforcing this legislation the following rules have been laid down for agents, and, so far as I know, have been substantially followed: First, to make no false statements in order to get evidence. It is, of course, a suppression of truth if the agent goes into an office and asks to see "the doctor," without disclosing the real object of his visit. Certainly he does not voluntarily say, "Doctor, I come to get evidence against you;" but yet his instructions are that even as to this he must answer truly all questions, notwithstanding that they may lead to the disclosure of his real errand. Secondly, to persuade no one to practise who is reluctant to do so. Thirdly, to report if there is reason to believe that the suspected person is ignorant of the law; so that notice may be sent to the offender. If after the arrest the society is satisfied that clemency is properly to be exercised, the prosecution is withdrawn upon defendant's stipulation to observe the law in the future. Under these rules, during the time that I have had any connection with the enforcement of these laws, we have fortunately escaped scandal; no agent has been proved a blackmailer; no innocent person, we hope, has been punished, and the enforcement of the law has been such, we trust, as to assure the officers of justice that only public aims have been kept in view, and that prosecution has never degenerated into persecution. The chief element in the efficacy of a law is, of course, the possibility of enforcing it; and that depends largely upon local sentiment. In this city little or no difficulty has been experienced in enforcing medical and dental laws. The police magistrates and grand juries have not been influenced by personal feelings, and the press has commended the laws and their administration. But in the rural districts there has been a different experience, which two instances will illustrate.

A certain man, having fallen into bad odor in Massachusetts, came to this city and obtained a position as an assistant by falsely pretending to have been graduated from the Boston Dental College. Compelled to leave New York, he went to Herkimer. A competitor in business reported him to the State society. He was notified to obey the law, and again tried in vain to pass examinations. This he never succeeded in doing, but being a man of plausible address he so worked upon the feeling of the neighborhood by representing himself as the victim of a rival's desire to monopolize the business of the town, that he not only managed, despite a strenuous charge of a judge of the Supreme Court, to escape indictment at the hands of three grand juries, the members of which by voting in his favor necessarily contravened their instructions and oath of office, but he even so obsessed the local editors that they took up the cudgels in his behalf, and one of them audaciously declared, in his editorial column, that although there was no doubt that the man was technically violating the law, nevertheless, no grand jury of that vicinage would ever indict him; an enunciation of rustic morals that a municipal journal, the New York *Times*, sharply scored. An even more striking instance of the willingness of grand jurors, under similar circumstances, to violate their oath of office and resolve themselves into Cadis, occurred at Warsaw in this State. Two dentists, graduates of colleges, established themselves in this town, and soon after a former resident of the place, who had been a practising wheelwright, returned from the West and also established a dental office. The strangers reported him to the grand jury; that intelligent body sent for the county clerk's register, and, finding that neither the informers nor the person informed of were registered, observed their oath by promptly indicting the strangers and violated it by dismissing the bill as to their friend and townsman. It has been said that the law is a school-master, and these instances go to show what a very large school exists in the rural districts.

*Disabilities.*—In this State, and the tendency throughout the country is to make the laws uniform, there are two preliminary requirements of license to practise dentistry: First, that the licenciate shall have passed satisfactory examinations to be evidenced by a diploma; secondly, that he shall register his name and qualifications in the clerk's office of the county wherein he practises. The object of this second requirement is to inform the public of the practitioner's authority to practise, and to render his punishment practicable in case his pretensions are false. Not only is the unlicensed practitioner liable to punishment criminally, but he is also under civil disability. He cannot maintain an action to recover his fees. He cannot maintain an action for slander should he be called a quack, for, in legal contemplation, he is one, no matter what his professional attainment may be. Should he be called as an expert in á trial, he may be prohibited by statute, as in Wisconsin, from so testifying; and even if allowed to testify, in the absence of a statutory prohibition his testimony would be regarded probably as of little weight. A case illustrating the point was that of Merville *vs.* Merville, tried in Herkimer on November 21, 1887. Plaintiff sued her brother-in-law for assault, alleging that he had knocked her tooth down her throat. Defendant denied the assault, and alleged that, while plaintiff herself was assaulting him and he was trying to keep her off, his finger unfortunately got into her mouth, which she promptly closed; that thereupon he naturally pulled the finger away sharply, and in so doing unwittingly extracted plaintiff's tooth with no other license than the right of self-defence. An important question then upon the trial, two years after the alleged assault, was this: Did defendant knock in or pull out plaintiff's tooth? Each side called an expert to maintain its contention. Defendant's expert, who had only studied in an office, testified that after two years it was impossible to tell from the appearance of the gum which theory of the assault was correct. On the other side, the man from Massachusetts previously mentioned testified favorably to the prosecution's theory; and qualified by swearing falsely that he had been graduated from the Boston Dental College. An examination of the dental register of Herkimer would have shown that neither expert was, at the time, registered, and cross-examination would have disclosed probably the falsity of the latter's statement in qualifying as an expert.

*What constitutes the practice of dentistry* must be in every case a question of fact for the jury. The word practice implies an habitual, customary course of action. But ordinarily, proof of a single extraction or treatment of a tooth, with the acceptance of a fee, has been held sufficient proof of practice; and even if the fee be not actually paid, it will suffice if the intent to receive it is clear. The exhibition of a sign, such as a golden tooth, a set of false teeth, or other indication that dental assistance can be had within, the giving of cards, and advertisements in newspapers, all go to establish the intent; and, under the North Carolina act, it was held sufficient proof of medical practice to show that the defendant held himself out as a physician and solicited patients. Under our statute, laboratory work, that part of dental work which is mechanical and no more, and is exercised upon inert matter only, does not constitute practice of dentistry, nor does assistance rendered to an operator by a student in his office for purposes of clinical instruction. Nor is it common sense to suppose that aid rendered by a layman to a licentiate in medicine or dentistry, or even in the absence of a licentiate, in case of emergency, should be considered practice within the meaning of the statute. Treatment of diseases of the mouth by a licensed physician or surgeon is declared by our statute not to be prohibited dental practice. But it is a question whether any of these exemptions are wise. They were not put in the law because regarded as necessary, but as mere tubs to the whales, who fancying them of importance could have defeated the law except for their presence in it. Their effect is chiefly harmful, in that they encourage unscrupulous men to believe that by hanging out the sign of "mechanical dentist," or by calling their unlicensed assistants "students," they may evade the provisions of the law. The question has arisen often,—

*Is Dentistry a Profession?*—To me it seems clear that it is not a separate profession, but a department of medicine, connoting by medicine both physic and surgery. But I am well aware that the contrary position has been taken both by dentists and laymen. That a dentist is a tradesman, an artisan, a medical man, are all propositions that have been argued in the courts. In the English case of Lee *vs.* Griffin (30 L. J. Q. B., 252), it was held that a contract to make a set of artificial teeth to fit the patient's mouth was a contract for the sale of a chattel required to be in writing under the statute of Frauds, and would not sustain an action for labor and materials. So in that case the dentist, whose patient died before the teeth were fitted, lost his action against her executors; and worst of all, Crompton, J., remarked in the case, "There can hardly be said to be more skill in fitting teeth than in fitting a pair of breeches." In Mississippi the court held, in 1856, in the case of Whitcomb *vs.* Reid (31 Miss., 567), that dental instruments were not embraced in the exemption of mechanical tools from levy under attachment, for the reason that dentistry is not a trade or handicraft, but a profession requiring "knowledge of the physiology of the teeth." In 1868, Chief Justice Cooley held, in the case of Maxon *vs.* Perrott (17 Mich., 332), that such instruments were exempt from levy not because a dentist is a mechanic, but because the calling is of a duplex nature and is mechanical so far as tools are concerned. The court said, "Indeed, dentistry was formerly purely mechanical, and instruction in it scarcely went beyond manual dexterity in the use of tools; and a knowledge of the human system generally, and of the diseases which might effect the teeth and render an operation important, was by no means considered necessary. Of late, however, as the physiology of the human system has become better understood and the relations of the various parts and their mutual dependence become more clearly recognized, dentistry has made great progress as a science, and its practitioners claim, with much justice, to be classed among the learned professions." Very recently, in a Missouri case (State *ex rel.* Flickinger *vs.* Fisher, 21 S. W., 446–593, 24 S. W., 167), the question arose before the Supreme Court of that State whether a practitioner of dentistry could, under the statute, claim exemption as a medical practitioner from jury service. Four judges constitute the first and three the second division of that court. The question came before the first division—one member being absent—in February, 1893, and they unanimously held the dentist to be a medical man; quoting in part an address of Dr. M. I. Davis, President of the American Medical Association, the court said, "While dentistry as an independent calling may have had a humble and comparatively recent origin, it has now become a very important branch of medical science. The fact that this branch of the medical profession has grown to such proportions as to have its own independent colleges and to confer its own degrees, and that it has become necessary that its practice should be regulated by statute, indicates the importance of the exercise of its functions to the public welfare. The fact that it is regulated in a separate article, and as an independent calling from that of M.D., does not in any manner affect the character of those functions." In December, 1893, the case again came up, and this time before the full bench, and a contrary opinion was given by four judges,—a majority,—the other three dissenting and adhering to their former opinion. In the course of the prevailing opinion it was said, somewhat flippantly for a judicial opinion, "Relator evidently feels unsteady on his logical legs if his sole reliance is to be on the statutory exemption heretofore noted, and so he resorts to the lexicographers and quotes from the Century Dictionary, where 'dentist' is thus defined: 'One whose profession it is to clean and extract teeth, repair them when diseased, and replace them, when necessary, by artificial ones; one who practices dental surgery or mechanical dentistry; a dental surgeon.' If he had delved more deeply into the science of definitions and had turned another page of the same work, he would have found 'Chiropodist: one who treats diseases or malformation of the hands or feet; especially a surgeon for the feet, hands, and nails; a cutter or extractor of corns and callosities; a corn-doctor.' So that if relator is exempt from jury duty because, as he says, he treats professionally diseases of the oral cavity, so also is his less pretentious professional brother, who with equal scientific skill treats diseases or malformations of the hands or feet and who is content to be dubbed corn-doctor." In Great Britain the Dental Act of 1878 (41 & 42 Vict., Ch. 33), places the practice of dentistry, like that of medicine, under the supervision of the General Council of Medical Education and Registration. The new French law confines the practice of dentistry to doctors of medicine or surgeon-dentists holding the government diploma. Why there should be any disposition on the part of dentists to separate themselves from other medical men and form a separate profession I fail to see; and in an article appearing in the *Medical Record* of December 4, 1886, entitled "Is Dentistry a Specialty of Medicine?" written before I had the honor to be retained by your State Society, I endeavored to point out what seemed to me to be the fallacy of an article in the same journal, on the 20th of the previous November, written by a former president of that Society, entitled "Dentistry not a Specialty of Medicine." The whole tendency of the day, and it is not in all respects a good one, is to medical specialization. Formerly we heard of physicians or surgeons; now we hear of gynæcologists, orthopedists, dermatologists, otologists, pretty much every sort of an ologist, including the stomatologist. And why should the last of them all, who, in so far as he is not an artisan or manufacturer, is very clearly a specialist in the study and treatment of maladies manifesting themselves in the human mouth, be otherwise than proud to be classed in the great body of medical men? Why should he not take the broad view of Celsus, that all branches of medicine are so interwoven that they cannot be separated, rather than the narrow view, begotten, I sometimes think, of injudicious vanity, that he should leave the society of his brethren and sit by himself in a corner? That the dentist's scientific work is medical work is recognized when we are compelled, in drafting and enforcing the dental law, to provide that the physician shall not be regarded, when doing oral work, as entering upon the forbidden field of dental practice. I have known physicians who antagonized the dental act for fear that it might be applied by narrow souls to prevent them from treating diseases of the mouth or to forbid a physician in rural districts from extracting a tooth; and I fear that there are some men with dental licenses who would be willing to see the law enforced in that spirit. But, fortunately, the standard of a profession is set by its leaders and not by its hangers-on, and the drift of dental legislation is towards educating first a physician, and then allowing him to specialize himself as a dentist. This, as it seems to me, is what should happen. First should come the broad foundation of a medical education; thereafter let the specialization follow upon that solid basis. I speak as a layman and, perhaps, impractically. But this much does seem certain to me, that if a dentist is to be permitted to treat disease as it is manifested in the human mouth, medical education should be required of him. If, on the contrary, his is to be a totally separate calling, he must not complain if he is grouped with craftsmen. This State has gone mad on providing for examinations and licenses for all sorts of callings. First the physicians, then the dentists, then the pharmacists, then the veterinaries, then the plumbers, and now in this year of grace the Senate has passed a bill incorporating chiropodists into the Pedic Society, with all the privileges of medical and dental societies, and another bill is introduced regulating the practice of horseshoeing, and it is proposed also to license accountants. Where is this sort of thing to stop; and is it not worth while for men whose common duties lie in treating bodily infirmities to come under common regulations? Let me apply one test to show wherein the trade and the profession differ, and then ask with which you will be grouped. The relation of tradesman and customer ends with the sale. That of artisan and employer with the job. Not so the relation of professional man and client. A priest, a lawyer, or a physician who, at any time, should disclose his knowledge gained in the confidential relation with the penitent, client, or patient, would be rankly dishonorable and tabooed among his fellows, but the merchant may discuss his sales, the plumber his pipes, and Trilby's foot is for all the world to see.

*The contractual relation* between dentist and patient, assuming that the dentist is a medical specialist, is the same as that which prevails between other medical men and their patients. Formerly the compensation of physicians and lawyers was in the nature of voluntary payment by the patient or client, known as the *honorarium.* There was no right of action to collect fees for their services. Apothecaries and surgeons might sue for fees, being regarded rather as tradesmen than professional men. The barrister, to-day in England, cannot sue for his fees; but since the passage of the Medical Act a physician may do so, providing he does not belong to a society of which the by-laws prohibit members from bringing such an action. In this country, however, the physician has always been regarded as entitled to recover the value of his services in an action at common law, and so as a rule has the dentist, although, as we have seen, it was once decided in an English case that a dentist who made a set of artificial teeth was not entitled to bring an action for his services, but only for the value of the goods sold and delivered. But that case would scarcely be followed in our courts as I think. Of course it is competent for a medical man as for any other person to enter into a special contract with his patients, even a contract of the "no cure, no pay" variety, which, however, is not favored in the law. In the absence of such a special agreement the contract on the part of the physician or surgeon and dentists is not to guarantee or insure a successful treatment of the case, but only, first, that he possess the reasonable degree of skill and learning ordinarily possessed by members of his profession qualified to practise in similar localities,—for the same degree of learning and skill is not required of a practitioner in a sparsely settled community that is demanded of one in large cities with clinical opportunities and access to libraries; secondly, that he will use reasonable and ordinary care and diligence in exercising his skill and applying his knowledge; and, thirdly, that he also use his best judgment. These rules are equally applicable to dentists as to other medical men; although it is conceivable that just as the courts hold physicians of different schools to responsibility according to the tenets of the so-called schools, in like manner they might, in the present nebulous condition of the judicial mind as to a dentist's classification, require of him a less degree of skill and knowledge in treating diseased conditions of the mouth and a higher degree of mechanical skill. Suppose, for example, that a dentist was sued for damages for malpractice in filling a tooth. It is obvious that the question may be twofold. First, whether the tooth should have been filled at all; whether as a matter of medical opinion its presence in the mouth was not a cause of nervous disturbances affecting the general health of the patient; and, secondly, whether the mechanical work of filling the cavity was properly performed. And in such a case, as I say, it might prove that one court, regarding the dentist as a medical man, might hold him to a higher degree of physiological knowledge, while another, regarding him as an artisan, might only require a proper degree of mechanical skill. But in the purely mechanical parts of the profession the same rules apply in construing the contract as are applicable in any action requiring manual or artistic skill, whether it be the painting of a portrait or the making of a suit of clothes. It is evident that the rules governing the liability for malpractice grow out of this rule regulating the contractual relation; for malpractice is nothing except failure, on the dentist's part, to perform his part of the contract, and he is, therefore, only guilty of malpractice when he treats a patient either without the average skill and learning required of his profession under like circumstances; or if possessed of such skill and learning, when he fails to exercise them with diligence and care; or when, exercising skill and learning with diligence, he fails to make proper exercise of his judgment. Where there is reasonable ground for error in judgment, as there must always be in doubtful cases, a mistake does not, of itself, constitute malpractice. The error must be so grave as to indicate that the defendant either lacked judgment sufficient to justify him in undertaking the case or that he acted rashly without due deliberation and thought. In a New York case (Keily *vs.* Colton, 1 City Ct., 439), it was said that a dentist owes the highest degree of skill and care to a patient under an anæsthetic; and that the fact that an operator suffers a tooth to slip down the throat of the unconscious patient is *prima facie* proof of negligence. Frequently anæsthetics operate in unforeseen ways owing to the personal idiosyncrasy of the patient, and it was held in Pennsylvania (Bogle *vs.* Winslow, 5 Phila., 136) that a dentist was only bound to look to the natural and probable effects of the anæsthetic. But it certainly does not seem a hard rule to require of a dentist administering anæsthetics the same degree of skill and knowledge required of a physician or surgeon under like circumstances; and this substantially was the view taken in the case last mentioned. If the dentist employ an inefficient assistant or let his student operate upon patients, he will be liable for their malpractice. Nor will the fact that the service was gratuitously rendered excuse negligence. If by his own fault the operator extract the wrong tooth, he is of course liable; but not so if the patient points out the wrong tooth, unless, perhaps, the condition of the teeth be such as to make the patient's mistake apparent. The patient's contractual obligation is to pay for the service rendered. And in a Vermont case (Gilman *vs.* Andrus, 28 Vt., 241), it was held that false teeth were necessaries to a married woman for which her husband would be compelled to pay.

The partnership relation is regulated by the same rules that obtain in any business, and is within the law merchant (Allen *vs.* Blanchard, 9 Cow., 631). A partner is liable only for acts of another if done within the scope of the mutual authority; as if he gives notes for supplies, etc. It is to be noted that a physician has been and a dentist may be held liable for the negligence of a partner; and this on the theory that each partner guarantees to the public that the one in charge brings to the business the requisite care, skill, and knowledge (Hyrne *vs.* Erwin, 23 S. C., 226; Whittaker *vs.* Collins, 34 Minn., 299). The imitation of another's sign is also regulated by the mercantile law (Colton *vs.* Thomas, 2 Brews., 308).

The *sanctity of the person* is protected by the law, and its violation punished. No liberty is excusable, however slight, not necessitated by the operation. The opportunities for improprieties are great when a patient is alone under anæsthetics; and, on the other hand, the possibilities of a blackmailing charge are equally great. In a Western State a physician called to a case of childbirth, who took a layman to carry his lantern and permitted him to come into the patient's room, the only one in the house, was mulct in damages (DeMay *vs.* Roberts, 46 Mich., 160). And a patient may not be made the subject of clinical demonstration against his will.

*Expert Testimony.*—The practice of calling experts in trials growing out of the common law right to call as a witness one specially skilled in an art or science has grown to such an extent that expert testimony is treated in separate volumes of the law libraries. It is possible here only to glance at one or two salient principles. It is a common fault of human nature that we espouse over zealously the cause in which we are interested. A witness is sworn to tell the truth, the whole truth, and nothing but the truth. Some witnesses try to obey their oath in all respects, so far as the court and the attorneys will permit them to do so. Many violate their oath in all respects. The honest expert tries to tell the truth and nothing but the truth in answering questions put to him. He is not always so desirous of telling the whole truth; so that it has come about of late years, especially in cases involving medical testimony, that expert witnesses are regarded in the light of special medical counsel, and many plans have been suggested for the modification of our system. At present the expert is generally examined upon a hypothetical question, framed either by himself or the counsel who calls him, and of course intended to bring out the facts favorable to his contention. He is cross-examined upon another hypothetical question framed by the counsel or expert on the other side; and too often intended to trip or muddle him. Occasionally he is bowled out of court in a few minutes by a clever cross-examiner, with questions having no bearing upon his scientific opinion. A notable instance of this was the confusion of a leading expert from another city who came here to testify in a famous case as to the diagnosis of morphine-poisoning, who narrated his experience humorously on his return home.

The cross-examination amounted to little more than this:

*Q.* When was your last case of morphine-poisoning?

*A.* About eight years ago.

*Q.* Were you certain it was morphine-poisoning?

*A.* The autopsy proved it.

*Q.* Do you always confirm your diagnosis by an autopsy?

*A.* No.

*Q.* When was your last case before that?

*A.* About fifteen years ago.

*Q.* And the case before that?

*A.* About twenty years ago.

*Q.* And do you, having had only three cases in twenty-five years and having needed an autopsy to confirm the diagnosis in one of them, mean to set your opinion up against that of men who have seen hundreds of cases in the same time?

By this clever stroke the force of the direct examination was destroyed in the minds of the jury; and a very modest and able man made to appear for the moment a very pretentious and ordinary person.

To remedy the evils of our present system and aid in the ascertainment of truth it has been suggested among other plans that there should be a trained body of experts appointed by the State who should inform the court in the premises. This, however, would be unsatisfactory under present conditions, since either party would still be entitled to call his own expert and to cross-examine the State's experts. Perhaps the best plan that has been suggested is that the experts on either side should examine the patient together and confer as to the results of their examination. This among fair-minded men of equal intelligence and skill ought to achieve in a majority of cases a substantial agreement, or at all events define clearly the scientific issues to be litigated. It is a generally accepted rule now that even in criminal cases expert testimony cannot be adduced merely by subpœna. A man may be compelled to tell in court what he actually knows of an occurrence, but not to put his scientific knowledge at the service of the parties without compensation. And perhaps it is out of this fact that much of the objection to expert testimony has grown, since the witness, especially if a man of weak moral nature, is necessarily biassed by his fee; particularly if it is contingent on the success of the party calling him. But when all this is said, the fact remains that truth is truth; and an honest man whose manner is not that of a partisan will generally be able to testify according to his knowledge whether we lawyers try to prevent him or not.

